# Features Associated with Visible Lamina Cribrosa Pores in Individuals of African Ancestry with Glaucoma: Primary Open-Angle African Ancestry Glaucoma Genetics (POAAGG) Study

**DOI:** 10.3390/vision8020024

**Published:** 2024-04-18

**Authors:** Jalin A. Jordan, Ebenezer Daniel, Yineng Chen, Rebecca J. Salowe, Yan Zhu, Eydie Miller-Ellis, Victoria Addis, Prithvi S. Sankar, Di Zhu, Eli J. Smith, Roy Lee, Gui-Shuang Ying, Joan M. O’Brien

**Affiliations:** Department of Ophthalmology, University of Pennsylvania, Philadelphia, PA 19104, USA; jalin.jordan@pennmedicine.upenn.edu (J.A.J.); ebdaniel@pennmedicine.upenn.edu (E.D.); yineng.chen@pennmedicine.upenn.edu (Y.C.); rebecca.salowe@pennmedicine.upenn.edu (R.J.S.); yan.zhu1@pennmedicine.upenn.edu (Y.Z.); eydie.miller@pennmedicine.upenn.edu (E.M.-E.); victoria.addis@pennmedicine.upenn.edu (V.A.); prithvi.sankar@pennmedicine.upenn.edu (P.S.S.); di.zhu@pennmedicine.upenn.edu (D.Z.); smiteli@pennmedicine.upenn.edu (E.J.S.); roylee@pennmedicine.upenn.edu (R.L.); gsying@pennmedicine.upenn.edu (G.-S.Y.)

**Keywords:** lamina cribrosa pores, primary open-angle glaucoma, African ancestry, stereo optic disc images

## Abstract

There are scarce data regarding the rate of the occurrence of primary open-angle glaucoma (POAG) and visible lamina cribrosa pores (LCPs) in the eyes of individuals with African ancestry; the potential impact of these features on disease burden remains unknown. We recruited subjects with POAG to the Primary Open-Angle African American Glaucoma Genetics (POAAGG) study. Through regression models, we evaluated the association between the presence of LCPs and various phenotypic features. In a multivariable analysis of 1187 glaucomatous eyes, LCPs were found to be more likely to be present in eyes with cup-to-disc ratios (CDR) of ≥0.9 (adjusted risk ratio (aRR) 1.11, 95%CI: 1.04–1.19, *p* = 0.005), eyes with cylindrical-shaped (aRR 1.22, 95%CI: 1.11–1.33) and bean pot (aRR 1.24, 95%CI: 1.13–1.36) cups versus conical cups (*p* < 0.0001), moderate cup depth (aRR 1.24, 95%CI: 1.06–1.46) and deep cups (aRR 1.27, 95%CI: 1.07–1.50) compared to shallow cups (*p* = 0.01), and the nasalization of central retinal vessels (aRR 1.33, 95%CI: 1.23–1.44), *p* < 0.0001). Eyes with LCPs were more likely to have a higher degree of African ancestry (q0), determined by means of SNP analysis (aRR 0.96, 95%CI: 0.93–0.99, *p* = 0.005 for per 0.1 increase in q0). Our large cohort of POAG cases of people with African ancestry showed that LCPs may be an important risk factor in identifying severe disease, potentially warranting closer monitoring by physicians.

## 1. Introduction

Glaucoma describes a group of eye diseases that progressively damage the optic nerve, subsequently causing the loss of visual fields. Glaucoma is the leading cause of irreversible blindness worldwide [1]. Primary open-angle glaucoma (POAG), the most common form of glaucoma, disproportionately affects African-ancestry individuals. These individuals are 4 to 5 times more likely to have POAG and up to 15 times more likely to experience vision loss from the disease when compared with European Americans [2,3,4]. Prior studies have investigated genetic and other risk factors that contribute to the disproportionate burden of POAG in African-ancestry populations [1,5,6]. One study suggested that structural differences in the optic nerve head (ONH) contribute to the increased prevalence and severity of POAG in these individuals [7]. It has also been demonstrated that African Americans with ocular hypertension have significantly larger optic discs, optic cups, and cup-to-disc ratios (CDR) when compared with other ancestral groups [8].

The lamina cribrosa (LC) is the porous collagen structure, located at the ONH, that provides entry and exit points for the blood vessels of the retina [9]. The LC is fixed between two pressurized compartments (ocular and craniospinal) and experiences a translaminar pressure difference (TPD) that makes it the primary site of retinal ganglion cell injury in TPD-related pathologies such as POAG; however, the extent to which these findings are linked to a higher risk of glaucoma, or its progression, is lacking [10,11,12]. Structural changes to the LC in glaucomatous eyes have been reported, including more frequent slit-shaped lamina cribrosa (LCPs), posterior displacement of the LC, and thinner LC [13,14]. The total number of LCPs can simultaneously increase as neural rim damage occurs in both POAG and normal-tension glaucoma [15]. LCPs in glaucomatous eyes have a more tortuous pathway when compared to healthy control eyes [16]. Additionally, LCPs are visible in 71% of POAG patients compared to 29.3% of controls [17]. Although several studies have characterized the changes to the LC in patients with glaucoma, few have investigated the associations between LCPs and demographic and ocular characteristics in African-ancestry populations [18,19,20]. Existing data show that African-ancestry individuals have a larger total LC area and a greater number of LCPs; however, there is no evidence linking these findings to a higher risk of glaucoma [5,16]. These studies have been limited by small sample sizes.

In this study, we investigated the prevalence of and factors associated with the presence of visible optic disc LCPs in a large cohort of African-ancestry individuals. Our aim was to discover the factors linked to visible LCPs in order to aid in identifying eyes with severe disease that potentially warrant closer monitoring by physicians.

## 2. Materials and Methods

### 2.1. Subject Recruitment and Clinical Assessment

The POAAGG study was an NIH-funded study that sought to elucidate the genetic architecture of POAG in an African-ancestry cohort. All subjects were ≥35 years and self-identified as Black (African ancestry, Afro-Caribbean, or African American). University of Pennsylvania (UPenn)-certified clinical research coordinators identified subjects during clinical visits to the Scheie Eye Institute, Perelman Center for Advanced Medicine, Philadelphia VA Medical Center, and Mercy Fitzgerald Hospital, as well as two neighboring ophthalmology clinics in Philadelphia, Pennsylvania (Windell Murphy, MD; Temple University) [21]. Additionally, a multimedia marketing campaign was employed with trusted African-ancestry community leaders to disseminate information and build trust in the community [22]. Exclusion criteria have been described previously [23].

At enrollment, subjects received an ophthalmic examination and onsite interviews. Subject data captured from the interview and examination were stored in the Research Electronic Data Capture (REDCap) database [24]. Demographic and lifestyle information were collected during the onsite interview. Family-related and past medical history were also obtained during the interview and extracted from patient medical records from the UPenn EPIC and MERGE systems.

Each subject was classified as a glaucoma case, suspect, or control by a fellowship-trained glaucoma specialist. In brief, cases were defined as having an open iridocorneal angle and characteristic optic nerve defects with corresponding visual field loss [23]. All subjects signed an informed consent form and provided a DNA sample. The University of Pennsylvania Institutional Review Board approved the study and the informed consent process, and the research adhered to the tenets of the Declaration of Helsinki.

### 2.2. Grading of Color Stereo Images of the ONH

Images were captured using the Topcon TRC 50EX retinal camera (Topcon Corp. of America, Oakland, NJ, USA) during the examination. Thirty-degree color stereo disc photographs of POAAGG subjects were uploaded to a secure server at the Scheie Image Reading Center at UPenn. The images were taken between 13 January 2004 and 25 June 2019, and were uploaded to the Scheie Image Reading Center server between 22 January 2016 and 20 April 2021. Grading occurred at the Reading Center between 6 June 2016 and 10 May 2021.

The images were analyzed by two non-physician POAAGG-certified graders to check for quantitative and qualitative phenotypic features. The grading process and its reliability have been described previously [25,26]. In brief, three non-physician graders trained by two glaucoma specialists evaluated images using a stereo viewer (Screen-Vu stereoscope, Eyesupply USA, Inc. Tampa, FL, USA). Differences in grader evaluations were reviewed and adjudicated by the Director of the Reading Center (ED), an ophthalmologist [26]. The qualitative grading process has been described in detail previously [26]. Briefly, two graders completed a standardized grading form for each stereo image pair (stereo images provide a 3D visual to qualitatively assess ocular features including cup depth) in order to determine image quality; disc shape; the shape of the cup; the presence of a tilted disc; disc hemorrhages and their location; arteriolar narrowing; both beta- and alpha-peripapillary atrophy; the narrowing of venules; the bayoneting of vessels; the pallor of the neural rim; the depth of the cup; rim plane position; the baring of the LC (grader determines if there are 0, ≤3, or >3 honeycomb-appearing visual pores); circumlinear vessels; sloping toward the outer rim of the disc; cilioretinal vessels; and gray crescents. The Reading Center Director also adjudicated disagreements between graders in terms of qualitative grading.

### 2.3. Re-Grading for LCPs

**Figure 1:**
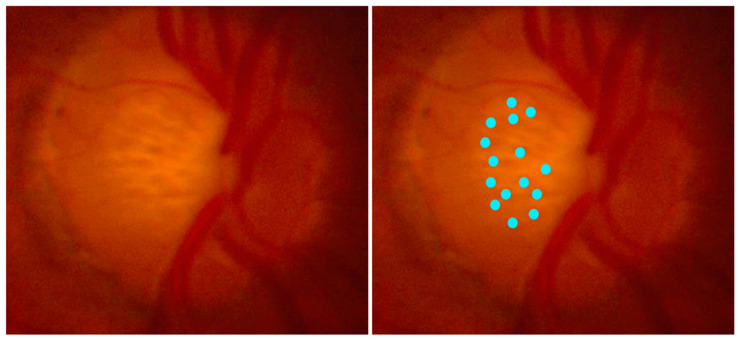
Fundoscopic image of optic nerve head with multiple LCPs (**left**). Blue dots highlighting the location of LCPs at the ONH (**right**).

In order to make the data more robust and prevent the misdiagnosis of LCPs, we re-graded images with LCPs where one or both original graders reported >3 pores or where both agreed that there were ≤3 (*n* = 2703). Between 31 March 2022 and 8 August 2022, a third, independent grader used an improved grading protocol with clear examples of LCPs and simplified the grading response to a binary value, with “Yes” for >3 pores and “No” for ≤3 pores. We used the presence of three pores as a threshold. This is because, typically, if there are at least three pores present, there will be multiple beyond that point (Figure 1). We created a spreadsheet containing the data regarding 3142 eyes, including 2703 eyes from regrading and 439 eyes where the original graders agreed the classification was “None”. Eyes marked “None” were excluded from the regrading due to higher degrees of intergrader reliability, as seen in previous papers [25]. We removed 199 images after being determined as ungradable, bringing the final total to 2943 eyes from 1551 patients for our analysis.

### 2.4. Specimen Collection and Ancestry Analysis

Peripheral blood or saliva samples were collected from all study subjects to perform genomic analysis [1,27]. Genotyping was conducted using the Multi-Ethnic Genotyping Array (MEGA)V2 (EX) consortium chip on the Infinium iSelect platform produced by Illumina FastTrack Services (Illumina, San Diego, CA, USA). Directly genotyped variants and samples were subjected to rigorous quality control, as detailed elsewhere [27].

After quality control, 1,108,459 SNPs were analyzed to determine continental ancestry using the 1000 Genomes Project version 5a dataset, as detailed in a previous publication [1]. This dataset contains five continental populations: African, Admixed American, East Asian, European, and South Asian. In brief, principal components were extracted from the genetic relationship matrix, using PLINK (version 1.90), in order to analyze continental ancestry. FastSTRUCTURE software (version 1.0) detected two-way admixture proportions among autosomal genotypes in POAAGG using two ancestral populations as a model [28]. This generated ancestral components q0 and q1, representing African and European ancestral components, respectively. The variables are continuous and sum to 1.0, with lower values of q0 denoting higher degrees of African ancestry and lower degrees of European ancestry [1]. In a previous publication, we found that the cases examined had a significantly lower mean value of q0 compared to controls, indicating a greater degree of African ancestry [1].

### 2.5. Genetic Analysis

A previous genome-wide association study (GWAS) identified potential causal loci for POAG in 11,275 individuals of African ancestry (6003 cases, 5272 controls). This mega-analysis included subjects from the African Descent and Glaucoma Evaluation Study (*n* = 1999) [29], the Genetics of Glaucoma in People of African Descent (GGLAD) consortium (*n* = 2952) [30], and the POAAGG study (*n* = 6324). A total of 46 risk loci were associated with POAG at a level of genome-wide significance. Replication analyses, trait colocalization analyses, and functional studies implicated three likely causal loci: rs1666698 located in *DBF4P2*, rs34957764 in *ROCK1P1*, and rs11824032 in *ARHGEF12*. For each SNP, we classified individuals based on the allele frequency (0, 1, 2) of risk gene. A total of 1966 eyes were evaluated in a univariable logistic regression analysis to determine the association between these SNPs and LCPs. Inter-eye correlation was accounted for using a GEE model.

### 2.6. Statistical Analysis

We used univariable Poisson regression models to evaluate the association between LCPs and demographic, ocular, clinical, and genetic characteristics among POAG cases. Cases were specifically chosen to understand LCPs as markers of a disease’s severity and its relationship with other glaucomatous features. Multivariable analyses were performed using a backward stepwise variable selection utilizing variables with *p* < 0.20, which were drawn from univariate analyses. Two separate multivariate analyses were reported (with and without consideration of clinical phenotype data) as there was a lack of detailed clinical phenotypic data for some POAG patients. All models accounted for inter-eye correlation by using generalized estimation equations (GEE). We reported risks ratios (RR), their 95%CI, and p-values for risk factors, drawing from univariable Poisson regression models and adjusted risk ratios (aRRs) from multivariable analyses. All statistical analyses were performed in SAS v9.4 (SAS Institute Inc., Cary, NC, USA).

## 3. Results

Demographic characteristics are described in Table 1. A total of 2117 eyes (71.9%) had LCPs. Individuals with a moderate BMI range of 25–30 had higher risk of LCPs, as opposed to values on both extremes (BMI < 25 and BMI ≥ 30) (*p* = 0.048). Higher degrees of African ancestry, as shown by lower values of the q0 variable, were associated with LCPs (RR = 0.97, 95%CI: 0.95–0.99, *p* = 0.006). Hypertension conferred a lower risk of LCPs (RR = 0.92, 95%CI: 0.87–0.98, *p* = 0.01).

The associations between qualitative ocular phenotypes and LCPs are shown in Table 2. LCPs were less likely to be present in eyes with an oval disc shape (RR = 0.92, 95%CI: 0.87–0.96, *p* = 0.003) compared to those with a round shape. Eyes with LCPs were also more likely to display the nasalization of the central vessels (RR = 1.39, 95%CI: 1.32–1.45, *p* < 0.0001), large CDR ≥ 0.9 (RR = 1.29, 95%CI: 1.20–1.38, *p* < 0.0001) or the bayoneting of vessels (RR = 1.24, 95%CI: 1.19–1.30, *p* < 0.0001), with moderate (RR = 1.45, 95%CI: 1.28–1.65, *p* < 0.0001) or deep cup depth (RR = 1.77, 95%CI: 1.56–2.01, *p* < 0.0001) compared to shallow depth, and bean pot/partial bean pot-shaped cups (RR = 1.59, 95%CI: 1.49–1.69, *p* < 0.0001) or cylinder-shaped cups (RR = 1.33, 95%CI: 1.25–1.42, *p* < 0.0001) compared to cone-shaped cups.

The associations of the available clinical phenotypes that were obtained within a ±90-day range of the image date are shown in Table 3. Eyes with LCPs had a significantly larger mean CDR (0.76 versus 0.62, *p* < 0.0001) and worse visual field mean deviation (−8.72 dB versus −6.35 dB, *p* = 0.007) than eyes without LCPs.

The results of our genetic variant analysis are included in Supplemental Table S1. The univariable analysis of genetic variants and LCPs in glaucoma cases did not unveil significant associations between the studied SNPs and LCPs.

We performed multivariate analyses, considering variables with *p* < 0.20 in the univariate analysis, in a backward variable selection model. Table 4 shows the results when all variables from the univariate analysis were included, resulting in a total of 654 eyes, of which 457 had LCPs (69.9%). CDRs ≥ 0.9 (aRR = 1.21, 95%CI: 1.11–1.33, *p* < 0.0001), th nasalization of central vessels (aRR = 1.34, 95%CI: 1.20–1.48, *p* < 0.0001), and lower q0 values (aRR = 0.96, 95%CI: 0.93–0.99, *p* = 0.005) were all significantly associated with LCPs. Cylindrical-shaped cups (aRR = 1.41, 95%CI: 1.24–1.61) and bean pot/partial bean pot cups (aRR = 1.36, 95%CI: 1.19–1.55) were more likely to have LCPs when compared to cone-shaped cups (*p* < 0.0001).

The analysis shown in Table 5 excluded q0, yielding 1187 total eyes, of which 856 (72.1%) had LCPs. The results were similar to those obtained when ancestry was included, which are shown in Table 5. LCPs were more likely to be present in eyes with a cylindrical cup shape (aRR = 1.22, 95%CI: 1.11–1.33) and bean pot/partial bean pot-shaped cups (aRR = 1.24, 95%CI: 1.13–1.36) when compared to the cone-shaped cups (*p* < 0.0001), as well as during the nasalization of the central retinal vessels (aRR = 1.33, 95%CI: 1.23–1.44, *p* < 0.0001), CDRs ≥ 0.9 (aRR = 1.11, 95%CI: 1.04–1.19, *p* = 0.005), and lastly with moderate (aRR = 1.24, 95%CI: 1.06–1.46) or deep cup depths (aRR = 1.27, 95%CI: 1.07–1.50) compared to shallow depths (*p* = 0.01).

## 4. Discussion

In this study, we investigated the prevalence and risk factors associated with the presence of visible LCPs in a large African-ancestry cohort. We showed that LCPs were associated with extremely large CDR, deep optic disc cups, cylindrical and bean pot-shaped cups, the nasalization of central retinal vessels, and higher degrees of African ancestry. Our univariate analysis initially suggested an association between LCPs and moderate BMI, but this was not supported in multivariate analysis. The impact of BMI on LC morphology remains unclear; a recent study found no difference in LC depth or thickness between obese and non-obese individuals [31].

We noted that several morphological changes to the optic cup were associated with the presence of visible LCPs. These LCP-associated cup features could be surrogates for the thinning of LC, the posterior displacement of LC, laminar deformation, and the curvature of LC, which have been described by Swept Source Optical Coherence Tomography [29]. Thinner LC is a risk factor that can cause normal-tension glaucoma suspects to convert to glaucoma cases [30]. LC thinning has also been associated with large CDRs and decreased vessel density in glaucoma suspects [32,33,34]. LC parameters such as cup depth, LC depth, prelaminar tissue thickness, and LC curvature index were associated with higher IOPs and thinner nerve fiber layers [35], as well as with a faster rate of RNFL loss [36]. Depth variations in the cup floor also differed between groups of normal subjects and glaucoma patients. This surface variability at the floor of the optic cup may represent a measurement of LC fragility that has been implicated, but not previously estimated, in glaucomatous eyes [37]. Glaucomatous eyes appear to undergo multiple changes in the LC, which may predispose patients to or be the result of other phenotypic changes.

Cone-shaped cups, used as a reference in this study, have a gradually sloping wall and may undergo the least deformation of the LC. Cylinder-shaped cups and bean pot-shaped cups possibly have more severe deformation of the LC. Although this has not been proven, longitudinal studies of glaucomatous eyes with imaging moralities such as SD-OCT should inform us in the future as to whether conical cups progress to cylinder-shaped cups and then to bean pot cups—and whether the LCPs start appearing at this point. The depth of the cups was independently associated with the presence of visible LCPs after adjusting for the shape of cups. OCT emerged as a comprehensive modality capable of more fully assessing LCPs. One such study showed an increased ratio of beam thickness to pore diameter and enhanced pore diameter variability in glaucomatous eyes compared to healthy controls [38]. To our knowledge, no study has compared the visibility of LCPs in OCT to traditional methods; however, OCT’s capability to assess microstructural features provides more robust data [32].

Visual field loss has been associated with elongated LCPs in glaucomatous patients; pores become more elongated and less circular with increasing field loss [39]. Small, round pores were associated with mild field loss, oval pores with moderate field loss, and striate or slit-shaped pores with advanced field loss [14]. We did not characterize the LCP shapes in this study, but did find a significant association between the presence of visible LCPs and worse visual fields in the univariate analysis.

We were unable to find an association between the three SNPs previously implicated in a large GWAS of POAG. Thus, our results suggest that these specific SNPs may not be determinants for the visibility of LCPs in African-ancestry patients. To our knowledge, no other study has explored genetic associations with visible LCPs. Future studies with larger sample sizes or additional variants may be warranted in order to explore potential associations more comprehensively.

A strong association was found between visible LCPs and the nasalization of central vessel trunk (CRVT) that had not been reported previously. The nasal displacement of CRVT has been reported as helping to predict the progression of central visual fields [40,41]. CRV has a nasally angled pathway through the LC [42], but it appears that the more nasally located CRVT in glaucoma is present in the prelaminar area and not in the LC deformation area [43].

We found that lower q0 values, which corresponded with higher degrees of African ancestry, were associated with the presence of visible LCPs. The optic disc features in blacks and whites with normal IOPs were different, with larger cup volumes and CDRs present in blacks [44]. In a large cohort of subjects with ocular hypertension, African Americans had significantly larger optic discs, optic cups, neuroretinal rims, and CDRs than other ancestral groups [8]. A number of studies on glaucoma subjects have shown that blacks display greater progression of glaucoma-related damage, a greater incidence of hemorrhage in and around the disc, earlier onset of POAG, greater prevalence and higher risk of IOPs, higher risk of reoperation after MIGS procedures, lower utilization of eye care services, and greater rates of blindness when compared to whites [45,46,47,48,49,50,51,52,53].

The relationship between morphological changes in the LC between African-descent (AD) and European-descent (ED) subjects was not consistent. While anterior lamina cribrosa surface depth (ALCSD) was found to be deeper in AD than ED glaucoma patients in a cross-sectional study [54], a different result was observed in a longitudinal study among glaucoma patients, where the migration of ALCSD was observed to be more posterior in ED compared to AD subjects [55,56]. Other studies showed that, in healthy individuals, African-descent groups had a greater acute posterior bowing of the LC and thinner LC [57,58]. Experiments on healthy eyes received from eye banks showed that, even though blacks had larger total LC area and a greater number of laminar pores than whites, the connective tissue proportion and pore size distribution in the laminae cribrosae of AD individuals were almost identical to those of ED individuals [59]. Ethnic differences in optic disc features in healthy eyes are not limited to blacks and whites, but also includes Chinese and Indian individuals, with Chinese people showing a deeper LC [60].

Our study has multiple limitations to note. There is variability in the treatment status and treatment type among cases, which could affect associations between LCPs and variables such as IOP. Next, the grading of cup features is subjective, and therefore less reliable when compared to objective processes. There were other features, such as axial eye length or other refractive measures, that we were unable to assess in this study; these features may have an effect on LCPs visibility and subsequent grader evaluation. We used double grading by experienced graders and the adjudication of discrepancies by an ophthalmologist to provide a robust evaluation. Additionally, it should be noted that the LC morphology continued to change, particularly in eyes with glaucoma, as deformation and remodeling of the neural and connective tissues of the optic nerve head took place [61]. Therefore, cross-sectional studies such as this are limited in our interpretation. Similarly, as our data were cross-sectional, we were unable to determine causality in terms of our significant associations. We lacked other ethnic groups to draw comparisons from, which may limit the generalizability of our study. We chose to focus exclusively on individuals of African ancestry with POAG in order to address the large disparities in terms of the prevalence of the condition and vision loss faced by this understudied population. Lastly, we opted to use data within a 90-day range of the subjects’ first imaging date. Many of the early images of eyes do not have corresponding phenotypic data in this range and as such, numerous data are in the “missing category”.

In conclusion, in our large cohort of glaucoma cases among individuals of African ancestry, we show that LCPs are associated with greater degrees of optic disc cupping and higher proportions of African ancestry. These results should inform those who manage patients with glaucoma, encouraging them to carefully follow patients who manifest LCPs in order to allow for timely treatment to prevent progression of the disease.

## Figures and Tables

**Table 1:** Univariable analysis of the association of demographic features and visible pores in the Lamina Cribrosa among POAAGG glaucoma cases (*n* = 2943 eyes).

		*n*	Visible Pores in the Lamina Cribrosa (*n* = 2117 Eyes)	RR (95%CI)	*p*-Value
Age (years)	≤60	682	511 (74.9%)	Ref	0.08
	(60, 70]	836	618 (73.9%)	0.99 (0.92, 1.06)	
	(70, 80]	880	606 (68.9%)	0.92 (0.85, 0.99)	
	≥80	545	382 (70.1%)	0.94 (0.86, 1.02)	
Sex	Male	1213	890 (73.4%)	Ref	0.22
	Female	1730	1227 (70.9%)	0.97 (0.92, 1.02)	
Body mass index	<25	689	502 (72.9%)	0.97 (0.91, 1.04)	0.048
	25–30	979	732 (74.8%)	Ref	
	≥30	1275	883 (69.3%)	0.93 (0.87, 0.99)	
Diabetes	No	1794	1312 (73.1%)	Ref	0.14
	Yes	1143	801 (70.1%)	0.96 (0.91, 1.01)	
	Missing	6	4		
q0 (per 0.1 increase in q0)		1807	1278 (70.7%)	0.97 (0.95,0.99)	0.006
Hypertension	No	641	490 (76.4%)	Ref	0.01
	Yes	2296	1623 (70.7%)	0.92 (0.87, 0.98)	
	Missing	6	4		
Family history of glaucoma	No	1191	847 (71.1%)	Ref	0.63
	Yes	1599	1153 (72.1%)	1.01 (0.96, 1.07)	
	Missing	153	117		
Alcohol use	No	1556	1114 (71.6%)	Ref	0.79
	Yes	1346	971 (72.1%)	1.01 (0.95, 1.06)	
	Missing	41	32		
Tobacco use	No	1339	982 (73.3%)	Ref	0.19
	Yes	1604	1135 (70.8%)	0.96 (0.91, 1.02)	
Previous glaucoma surgery	No	2067	1497 (72.4%)	Ref	0.43
	Yes	868	614 (70.7%)	0.98 (0.92, 1.04)	
	Missing	8	6		

**Table 2:** Univariable analysis of the associations between disc features determined by central reading center and visible pores in the Lamina Cribrosa among POAAGG glaucoma cases (*n* = 2943 eyes).

		*n*	Visible Pores in the Lamina Cribrosa (*n* = 2117 Eyes)	RR (95%CI)	*p*-Value
Disc shape	Round	1247	940 (75.4%)	Ref	0.003
	Oval	1592	1102 (69.2%)	0.92 (0.87, 0.96)	
	Other	10	6 (60.0%)	0.80 (0.45, 1.42)	
	Missing	94	69		
Cup disc ratio	<0.9	1003	683 (68.1%)	Ref	<0.0001
	≥0.9	268	235 (87.7%)	1.29 (1.20, 1.38)	
	Missing	1672	1199		
Shape of cup	Conical	1107	645 (58.3%)	Ref	<0.0001
	Cylindrical	1241	965 (77.8%)	1.33 (1.25, 1.42)	
	Bean pot/partial bean pot	395	365 (92.4%)	1.59 (1.49, 1.69)	
	Other	14	8 (57.1%)	0.98 (0.62, 1.55)	
	Missing	186	134		
Cup depth	Shallow	317	154 (48.6%)	Ref	<0.0001
	Moderate	1737	1225 (70.5%)	1.45 (1.28, 1.65)	
	Deep	704	605 (85.9%)	1.77 (1.56, 2.01)	
	Missing	185	133		
Tilted disc	No	2547	1832 (71.9%)	Ref	0.80
	Yes	275	200 (72.7%)	1.01 (0.93, 1.10)	
	Missing	121	85		
Disc hemorrhage	No	2802	2017 (72.0%)	Ref	0.29
	Yes	48	31 (64.6%)	0.90 (0.73, 1.11)	
	Missing	93	69		
Arteriole narrowing	No	2802	2010 (71.7%)	Ref	0.22
	Yes	48	38 (79.2%)	1.10 (0.95, 1.28)	
	Missing	93	69		
Beta parapapillary atrophy	No	856	627 (73.2%)	Ref	0.36
	Yes	2087	1490 (71.4%)	0.97 (0.92, 1.03)	
Venule narrowing	No	2806	2016 (71.8%)	Ref	0.90
	Yes	44	32 (72.7%)	1.01 (0.84, 1.21)	
	Missing	93	69		
Bayonetting	No	2462	1703 (69.2%)	Ref	<0.0001
	Yes	481	414 (86.1%)	1.24 (1.19, 1.30)	
Nasalization of central vessels	No	1751	1095 (62.5%)	Ref	<0.0001
	Yes	1095	949 (86.7%)	1.39 (1.32, 1.45)	
	Missing	97	73		
Pallor of the neural rim	No	2730	1958 (71.7%)	Ref	0.37
	Yes	119	90 (75.6%)	1.05 (0.94, 1.18)	
	Missing	94	69		

**Table 3:** Univariable analysis of the association of clinical measures and visible pores in the Lamina Cribrosa among POAAGG glaucoma cases (*n* = 2943 eyes).

	Visible Lamina Cribrosa Pores (*n* = 2117 Eyes)	No Visible Lamina Cribrosa Pores (*n* = 826 Eyes)	*p*-Value
Highest IOP (mmHg)			
N	1149	441	0.46
Mean (SD)	19.15 (6.56)	19.47 (6.76)	
Range	(2.00, 56.00)	(7.00, 52.00)	
Central corneal thickness (µm)			
N	869	332	0.28
Mean (SD)	533.43 (38.34)	536.71 (40.06)	
Range	(420.00, 690.00)	(433.00, 659.00)	
Cup disc ratio			
N	918	353	<0.0001
Mean (SD)	0.76 (0.15)	0.62 (0.20)	
Range	(0.10, 1.00)	(0.10, 1.00)	
Visual acuity (logMAR)			
N	872	328	0.56
Mean (SD)	0.31 (0.66)	0.29 (0.62)	
Range	(−0.12, 5.00)	(−0.12, 6.00)	
Nerve Fiber layer thickness (µm)			
N	410	159	0.16
Mean (SD)	73.38 (14.69)	75.38 (12.38)	
Range	(38.00, 120.00)	(46.00, 100.00)	
Visual field MD			
N	489	200	0.007
Mean (SD)	−8.72 (9.59)	−6.35 (8.71)	
Range	(−33.15, 22.08)	(−32.00, 21.01)	

**Table 4:** Multivariable analysis of the associations between features and visible pores in the Lamina Cribrosa among POAAGG glaucoma cases with complete data that included q0 (*n* = 654 eyes).

		*n*	Visible Pores in Lamina Cribrosa Present (*n* = 457 Eyes)	aRR (95%CI)	*p*-Value *
Cup Disc Ratio	<0.9	526	342 (65.0%)	Ref	<0.0001
	≥0.9	128	115 (89.8%)	1.21 (1.11, 1.33)	
Shape of cup	Conical	277	149 (53.8%)	Ref	<0.0001
	Cylindrical	273	214 (78.4%)	1.41 (1.24, 1.61)	
	Bean pot/partial bean pot	100	92 (92.0%)	1.36 (1.19, 1.55)	
	Other	4	2 (50.0%)	1.07 (0.38, 3.01)	
Nasalization of central vessels	No	387	225 (58.1%)	Ref	<0.0001
	Yes	267	232 (86.9%)	1.34 (1.20, 1.48)	
q0 (per 0.1 increase in q0)		645	457 (69.9%)	0.96 (0.93, 0.99)	0.005

* Using GEE model to account for inter-eye correlation. The multivariable model considers all risk factors with *p* < 0.20 in the univariable analysis in Table 1 and Table 2. The backward variable selection was applied to reach the final multivariable model.

**Table 5:** Multivariable analysis of the associations between disc features and visible pores in the Lamina Cribrosa among POAAGG glaucoma cases with complete data that did not include q0 (*n* = 1187 eyes).

		*n*	Visible Pores in Lamina Cribrosa (*n* = 856 Eyes)	aRR (95%CI)	*p*-Value *
Cup Disc Ratio	<0.9	945	644 (68.1%)	Ref	0.005
	≥0.9	242	212 (87.6%)	1.11 (1.04, 1.19)	
Shape of cup	Conical	525	317 (60.4%)	Ref	<0.0001
	Cylindrical	480	372 (77.5%)	1.22 (1.11, 1.33)	
	Bean Pot/Partial Bean Pot	174	162 (93.1%)	1.24 (1.13, 1.36)	
	Other	8	5 (62.5%)	1.06 (0.65, 1.72)	
Cup depth	Shallow	167	86 (51.5%)	Ref	0.01
	Moderate	735	520 (70.7%)	1.24 (1.06, 1.46)	
	Deep	285	250 (87.7%)	1.27 (1.07, 1.50)	
Nasalization of central vessels	No	673	405 (60.2%)	Ref	<0.0001
	Yes	514	451 (87.7%)	1.33 (1.23, 1.44)	

* Using GEE model to account for inter-eye correlation. The multivariable model considered all features with *p* < 0.20 in the univariable analysis in Table 1 and Table 2. The backward variable selection was applied to reach the final multivariable model.

## Data Availability

The original contributions presented in the study are included in the article/Supplementary Materials, further inquiries can be directed to the corresponding author/s.

## Supplementary Materials

The following supporting information can be downloaded at: https://www.mdpi.com/article/10.3390/vision8020024/s1, Supplemental Table S1. Univariable analysis of the association of genetic variants and visible pores in the Lamina Cribrosa among POAAGG glaucoma cases (*n* = 1966 eyes).
