# Supplementary material for: Features Associated with Visible Lamina Cribrosa Pores in Individuals of African Ancestry with Glaucoma: Primary Open-Angle African Ancestry Glaucoma Genetics (POAAGG) Study
Source: Vision (Basel). 2024 Apr 18;8(2):24. doi: 10.3390/vision8020024 (PMC11036295; doi:10.3390/vision8020024)
Supplement: Supplementary file 1 [file vision-08-00024-s001.zip › vision-2909059-supplementary.pdf]

Supplementary Materials: Features Associated with Visible Lamina Cribrosa Pores in Individuals of African Ancestry with Glaucoma: Primary Open-Angle African Ancestry Glaucoma Genetics (POAAGG) study

Supplemental Table S1. Univariable Analysis for association of genetic variants and visible pores in the Lamina Cribrosa among POAAGG glaucoma cases (N=1966 eyes)

Variants	Allele Frequencies	Without LCP	With LCP	OR (95% CI)	P-value
**rs1666698_ALT**	0	31 (36%)	55 (64%)	Ref	-
	1	223 (28%)	570 (72%)	1.44 (0.90, 2.30)	0.13
	2	302 (28%)	785 (72%)	1.47 (0.92, 2.32)	0.10
**rs11824032_ALT**	0	307 (28%)	799 (72%)	Ref	-
	1	220 (29%)	536 (71%)	0.94 (0.77, 1.14)	0.52
	2	29 (28%)	75 (72%)	0.99 (0.64, 1.54)	0.98
**rs34957764_ALT**	0	500 (28%)	1292 (72%)	Ref	-
	1	56 (32%)	118 (68%)	0.82 (0.58, 1.14)	0.23
	2	0	0	-	-
